# Seroprevalence of Neutralizing Antibodies against Japanese Encephalitis Virus among Adolescents and Adults in Korea: A Prospective Multicenter Study

**DOI:** 10.3390/vaccines8020328

**Published:** 2020-06-19

**Authors:** Byung Ok Kwak, Young Se Kwon, Young Jin Hong, Chung Hyun Nahm, Woori Jang, Young Uh, Yong Gon Cho, Jimyung Kim, Myungshin Kim, Dong Hyun Kim

**Affiliations:** 1 Department of Pediatrics, Hallym University Kangnam Sacred Heart Hospital, Seoul 07441, Korea; qquack00@daum.net; 2 Department of Pediatrics, Inha University School of Medicine, Incheon 22332, Korea; ysped@inha.ac.kr (Y.S.K.); hongyjin@nate.com (Y.J.H.); 3 Department of Laboratory Medicine, Inha University School of Medicine, Incheon 22332, Korea; nahm@inha.ac.kr (C.H.N.); jangwr@inha.ac.kr (W.J.); 4 Department of Laboratory Medicine, Yonsei University Wonju College of Medicine, Wonju 26426, Korea; u931018@yonsei.ac.kr; 5 Department of Laboratory Medicine, Chonbuk National University Medical School and Hospital, Jeonju 54907, Korea; choyg@jbnu.ac.kr; 6 Department of Laboratory Medicine, Chungnam National University Hospital, Daejeon 35015, Korea; jmkim@cnuh.co.kr; 7 Department of Laboratory Medicine, College of Medicine, The Catholic University of Korea, Seoul St. Mary’s Hospital, Seoul 06591, Korea; microkim@catholic.ac.kr

**Keywords:** Japanese encephalitis, seroprevalence, neutralizing antibodies, Korea

## Abstract

The immunization schedule for the Japanese encephalitis (JE) vaccine in Korea is a two-dose primary series at 12–24 months of age, followed by booster doses 12 months after the second dose and at the ages of 6 and 12 years. Although the number of JE cases has markedly decreased after the universal vaccination program, JE predominantly occurs in adults. The aim of this study was to assess the age-specific prevalence of the JE-neutralizing antibody (NTAb) among adolescents and adults in Korea. A total of 1603 specimens were collected from a healthy Korean population above 15 years old in five provinces. The JE-NTAb titers were measured with the pseudotyped virus assay and considered to be positive at ≥ 1:50. The seropositivity of JE-NTAb was the highest in the 15–29 years category (>95%) and gradually began to decrease in the age group of 30–44 years (89.42%). The lowest and second lowest JE-NTAb seropositive rates were observed among those aged 70 years or older (59.77%) and those aged 55–59 years (75.24%), respectively. Subjects from Seoul exhibited the highest JE-NTAb titer in all age groups compared to other provinces. In conclusion, the JE-NTAb seropositive rates and titers have maintained appropriate levels in the general Korean population. We propose that adult immunization and boosters at 12 years of age against JE are not strongly recommended in Korea.

## 1. Introduction

Japanese encephalitis (JE), a mosquito-borne flavivirus infection, is the leading cause of severe viral infection of the central nervous system in Asian children [1]. JE viruses are transmitted by bird-biting mosquitoes, especially mosquitoes from the *Culex* genus. There is no specific antiviral treatment for JE, and vaccination is the single most important control measure [2]. JE spread throughout Asia, but national immunization programs and urban development in the 1960s led to the near elimination of JE in Japan, Korea, Singapore, and Taiwan. However, JE remains endemic in much of the rest of Asia [3]. The virus was isolated in the 1930s, and the first inactivated mouse brain-derived vaccines were produced in the same decade [4]. For many years, only the inactivated JE vaccine produced in mouse brain was available in developed countries. Although at least eight types of JE vaccines are produced and used in several countries currently, the inactivated mouse brain vaccine still remains one of the principal vaccines. The immunization schedule for JE varies by nations. Primary vaccination is recommended at 18 months of age in Thailand, 15 months in Taiwan, 36 months in Japan, and 12 to 23 months in Korea [5].

Since the JE vaccine was first introduced in Korea in 1967, the incidence rate has decreased from 12,055 cases (mean annual incidence rate of 6.04/100,000 persons) in 1961–1967 to 3783 cases (mean incidence of 0.67/100,000) in 1968–1983 [6]. Following the last epidemic in 1982–1983, mandatory vaccination was implemented for all children aged 3–15 years annually until 1994 [7]. Thereafter, the vaccination schedule has changed two times in 1995 and 2000, and now a revised schedule of a two-dose primary series and three boosters is recommended [5,7]. As a result, JE was nearly eliminated, and only 55 cases (mean incidence of 0.004/100,000) were reported in 1984–2009 [6]. However, JE has reemerged since 2010 (26 cases), and the annual incidences of JE have increased to be highest in 2015 (40 cases) [8]. During 2010–2015, 129 JE cases were reported in Korea; the median age of the patients was 53 years, and patients <19 years of age accounted for only 3.1% of cases. These findings indicated that reemerging JE predominantly affected unvaccinated adults >40 years of age, and shifts in age distribution toward older groups might be due to the universal vaccination program. Similar findings were observed in nearby countries. In Japan, following the largest epidemic of approximately 2000 cases in 1965, the annual number of cases has dramatically decreased, and only less than 10 cases have been reported annually since 1992 [9]. During the period 1982–2004, 361 JE cases were reported, and most of the patients were 40 years old or older, with a peak in the 60–69 year age group. JE predominantly occurred in unvaccinated populations. In Taiwan, pediatric JE cases have markedly decreased since the vaccination, and a shift in JE cases from young children to adults has also occurred [10,11].

Therefore, it is necessary to assess the age-specific immunity to JE to make an appropriate vaccination policy to control JE. There have been studies on the immunogenicity of the JE vaccine for children [12,13], but there is a lack of prospective studies on JE-neutralizing antibodies (NTAbs), which were conducted on the general population in Korea. Moreover, most of the studies on the seroprevalence of JE in Korea were conducted between the 1960s and 1990s [14], and these studies were confined to small local areas. The aim of this study was to investigate the age-specific prevalence of JE-NTAbs among the general population above 15 years old in Korea.

## 2. Materials and Methods

### 2.1. Study Design and Participants

**Figure 1:**
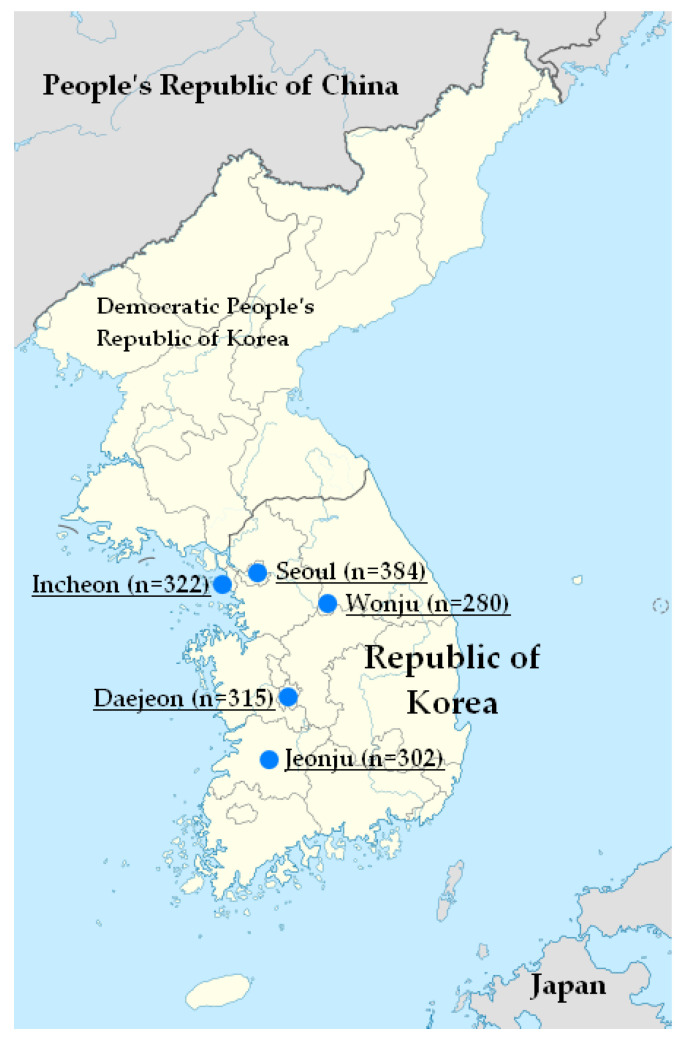
Geographical distribution of the study population.

This multicenter and prospective study was conducted in Korea between March 2012 and August 2012. The 1603 specimens were collected from clinically healthy adolescents and adults above 15 years old in five geographically distant hospitals (The Catholic University of Korea, Seoul St. Mary’s Hospital, Wonju Severance Christian Hospital, Inha University Hospital, Jeonbuk National University Hospital, and Chungnam National University Hospital) in Korea—Seoul as inland capital, Wonju as mountainous terrain, Incheon as a port city, Jeonju as plain ground, and Daejeon as middle inland (Figure 1). Blood samples were collected from each of more than 100 specimens at every age from the age of 15 to 19 years and at every 5 ages of interval from the age group of 20 to 69 years; 87 specimens were obtained over 70 years of age. The subjects with impaired immunologic function, malignancy, blood derivative use, immunosuppressant use, or other immune-modifying drug use were excluded. All the samples were obtained after written informed consent was obtained from each participant. The study protocol was approved by the Institutional Review Board of Inha University Hospital (IUH-IRB-12-09-088), as well as an independent review board at each study site.

### 2.2. Neutralizing Antibody Titers Using Pseudotyped Virus Assay

Serum was obtained from each subject and stored at −20 °C to −70 °C in a freezer until the measurement of the JE-NTAb titers. All the serum samples were sent to KR Biotech Co., Korea, and the NTAbs were measured using a pseudotyped virus (PV) assay. Although the plaque reduction neutralization test (PRNT) has been known as the standard method for detecting and quantifying NTAbs against the JE virus [14], in this study, a PV assay was used to measure NTAb titers due to limited expertise in growing and titrating JE virus plaques for investigating a large number of samples. The method of PV assay was described in a previous study [15]. In brief, the pseudotyped JE virus containing the env gene of the JEV NK strain was generated using MuLV packaging system. Virus titers were determined in the Vero cells by infecting with the virus and staining with X-Gal solution. The X-Gal-stained blue cells were manually counted using an inverted microscope. For the neutralization assay, the Vero cells were dispensed into 96-well plates and incubated for 16 h at 37 °C. Equal amounts of the diluted serum sample and diluted JEV-PV were mixed and incubated at 37 °C for 1 h. Thereafter, 100 mL of the neutralized liquid was inoculated into three wells per dilution and allowed to adsorb for 36 h at 37 °C/% CO_2_, and then X-gal staining was performed. The neutralizing titer was determined by testing serially diluted sera relative to the concurrently tested control serum prepared from normal mice. The neutralization titer was expressed as the maximum serum dilution yielding a 50% reduction in virus (PV_50_), and a titer equal or greater than 1:50 was considered to be seropositive. The comparison of the PV assay against the conventional PRNT has been performed and published previously [15].

### 2.3. Statistical Analyses

All of the statistical data were calculated by using SPSS version 17.0 (SPSS Inc., Chicago, IL, USA). The seropositive rates and titers of JE-NTAbs were calculated with 95% confidence intervals (CIs). An ANOVA and the Holm–Bonferroni method were used to compare geographical differences in the NTAb titers. To verify that the PV assay and PRNT were associated, we performed a linear regression analysis using bootstrapping. A *P* value of < 0.05 was considered to be statistically significant.

## 3. Results

### 3.1. Study Population

There was a total of 1603 participants, including 384 subjects from Seoul, 280 subjects from Wonju, 322 subjects from Incheon, 302 subjects from Jeonju, and 315 subjects from Daejeon. The distribution by age group in the five provinces is shown in Table 1.

### 3.2. Seropositive Rates of JE-NTAb Using PV Assay

**Figure 2:**
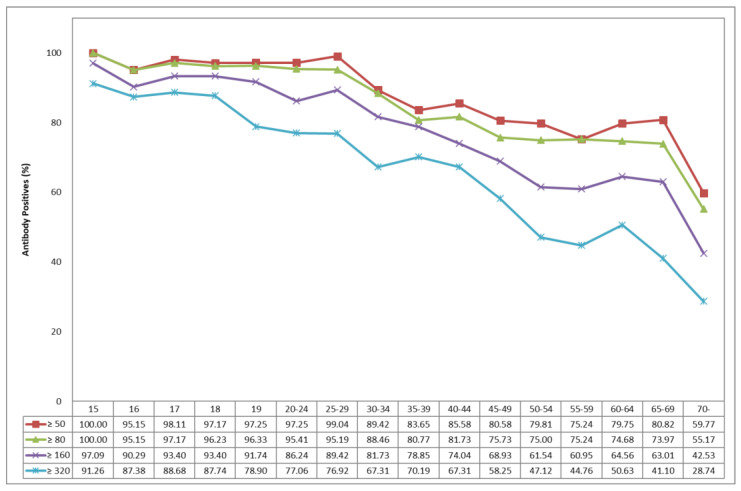
Age-specific seropositive rates of neutralizing antibody to Japanese encephalitis.

The age-specific seropositive rates of JE-NTAb in the Korean population over 15 years of age are presented in Figure 2. The seropositive rate was more than 95% for the age of 15 to 29 years and gradually began to decrease for the age of 30 to 54 years, reaching 75.24% for the age of 55 to 59 years, 80.82% for the age of 65 to 69 years, and 59.77% above 70 years old. For comparison, the results using the PRNT assay are displayed in Figure S1.

Subjects aged 15–19 years might have been fully vaccinated. Subjects aged 20–44 years who were born between 1968 and 1992 should have been vaccinated including annual boosters during their childhood. Subjects aged 45–59 years who were born between 1953 and 1967 may have been vaccinated, but there was a limited quantity of the vaccine after its first introduction in 1967. The subjects aged 60 years above who were born before 1952 were never vaccinated in childhood because the JE immunization program had not yet been introduced [6].

In the geographical results, the highest titer of JE-NTAb in the Seoul population was reported among all age groups, the lowest titer in Incheon was reported for the age group of 15 to 44 years, and in Jeonju the lowest titer was reported for the age group above 45 years, which were statistically significant (*p* < 0.05) (Table 2).

### 3.3. Model for Validation between PV Assay and PRNT

**Figure 3:**
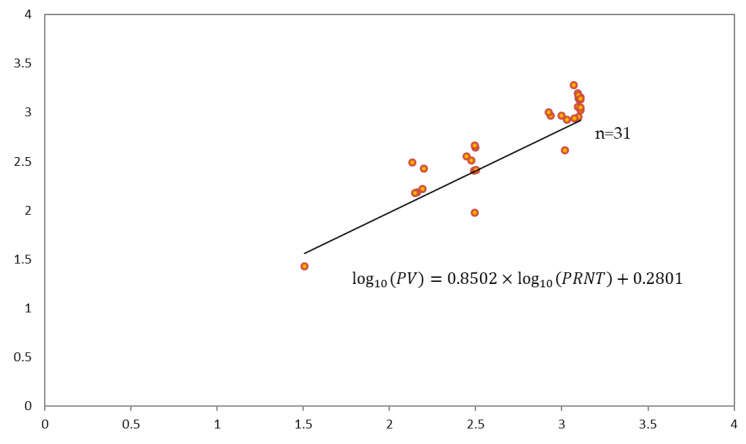
Regression model between the pseudotyped virus (PV) assay and the plaque reduction neutralization test (PRNT). log_10_(PV) = 0.2801 + 0.8502 × log_10_(PRNT).

Estimating the regression coefficients using 100,000 bootstrapping for 40 samples resulted in a higher mean value of the determinant, with 24 samples at 0.6058. Calculating the confidence intervals of 95% of the estimated coefficients, log_10_(PRNT) included 1 with 0.6267 to 1.0737 and the intercept included 0 with −0.3531 to 0.9133. According to the simple regression model analysis of this study, the r value was 0.778 for R^2^ = 0.6058, and the regression equation (Figure 3), excluding nine samples that exceeded the threshold of external standardized residuals for regression lines using bootstrapping between NTAb titers using the PRNT and PV assays, is shown below.

## 4. Discussion

The results of this study showed that the seropositive rate was more than 95% in 15–29 year age group and gradually began to decrease from the ages above 30 years to 75.24% in the 55–59 age group, followed by 80.82% in the 65–69 age group, and 59% in the 70 or older age group. The seropositivity and titers for NTAbs to JEV revealed appropriate levels for all age groups above 15 years old in Korea.

There have been noticeable changes in the age-specific JEV seroprevalence over time in Korea [14]. In 1946, seropositivity was 51% in the 1–10 years age group, 79% in those aged 11–20 years, and 94% in those over 61 years of age [16]. In the 1970s, the seropositivity in children and adolescents was low (10–59%) [17,18,19,20,21,22]. The seropositivity in this group increased to 90–92% in 1984–1985 [23], and increased further to 98% in our study conducted in 2012. On the other hand, the seroprevalence among adults has been maintained at a high level. In another Korean study, conducted in 2010 using 945 samples from adults aged 30–69 years in 10 provinces, the overall seropositive rate was 98.1%, and no age-specific difference was found [6]. In our study, the seropositive rates among adults aged 30–49 years and 50–69 years were between 81% and 89% and 75% and 81%, respectively. PRNT was used to measure NTAbs to JEV in the previous study, and differences in assays with differing cut-off thresholds may have affected the results. Moreover, environmental factors including residences, sanitary conditions, occupations, and vaccination statuses may have contributed to the differences between the two studies.

Our results differ from previous studies conducted in Japan [9] and Taiwan [10,11], where the vaccination policy spanned 40 years. In Japan in 2004, those aged 25–64 years and 70 years or more had a seropositive rate of between 20% and 70%, and the rate was lowest in the population 45–49 years of age [9]. This population was not vaccinated against JEV, nor naturally infected at a high rate as in the elderly population. In Taiwan, a similar pattern of the lowest JE-NTAb-seropositive rate (54%) was observed among adults born between 1963 and 1975, who generally received two or three doses of the vaccine and were administered the last booster dose more than 20 years ago [10]. The high JE-NTAb seropositive rates were observed in the oldest unvaccinated population (86%) and in the youngest population born between 1981–1986, who received four doses 10–15 years ago (74%). In both of these countries, a relatively low JE seroprevalence (NTAb seropositive rate of <50%) was observed in at least one age group—those aged 30–59 years in Japan in 2004 [9], and those aged 27–39 years in Taiwan in 2002 [10].

In our study, a gradual decrease in JE seroprevalence was observed in the 30–59 age groups, but there was no age group with a seropositive rate below 50%. The other Korean study also presented the overall seropositive rate of 98% in the adult population aged 30–69 years [6]. The high prevalence in Korea may be explained by the comparative magnitude of the JE epidemics and extensive vaccination program. In Korea, three large epidemics with 1000–3500 cases were reported in 1961–1968, and the latest epidemic in 1983 involved 139 reported cases [7]. The repeated and large JE epidemics within a short period may have resulted in the long-term maintenance of neutralizing antibodies in a high percentage of the elderly unvaccinated population in Korea. In addition, the mandatory vaccination program for all children aged 3–15 years annually was introduced in the 1980s, and it could have contributed to the high positive rates of neutralizing antibodies in the Korean population [6,7]. The revised JE vaccination schedule in Korea involved a two-dose primary vaccination from 12–24 months and three boosters [5]. In Japan, a two-dose primary vaccination at the age of 3 and two boosters at the age of 4 and 9–13 years are recommended [9]. The third booster immunization at 14–15 years was terminated as of 2005. In Taiwan, a two-dose primary vaccination is administered at 15 months old, and two booster doses are administered at 27 months old and in first grade of elementary school [10]. Based on the high seropositivity in our study compared to those of Japan and Taiwan, a final booster immunization at 12 years of age is not strongly recommended in Korea. Continuous surveillance on the seroprevalence of JE and JE cases is required to establish a proper immunization schedule in Korea.

In our study, NTAb titers differed by region. Seoul (a metropolitan city) showed the highest titer among all age groups, which is consistent with the previous study [6]. Although specific information on vaccine coverage was not obtained in this study, the high vaccination rate in Seoul compared to other provinces may have caused this result. In contrast, the lowest titer was observed in Incheon for the age group of 15 to 44 years and Jeonju for the age group above 45 years (*p* < 0.05). The vaccine coverage rate; subject’s occupation; and environmental factors related to virus exposure, such as sanitary conditions and pig farms, may have affected this difference. Further study is required to identify this geographical difference in JE-NTAb titers.

The strengths of this study include the fact that we prospectively investigated the seropositive rates and NTAb titers of JEV in more than 1600 samples collected from the general population above 15 years old in various provinces in Korea. Moreover, we evaluated the correlation between the PV assay and PRNT and validated the PV assay as an alternative method for measuring JE-NTAb titers. Our study has some limitations. First, information on the individual vaccination status, location of residence, occupation, and travel history was not available, and we could not determine other factors which may have influenced the seropositive rates. Second, we did not trace the kinetic waning of anti-JEV antibodies in each subject, but instead compared the seroprevalence by age group. Third, the study site was limited to Seoul, Wonju, Incheon, Jeonju, and Daejeon, and thus the results cannot be representative of the entire country. However, we included various provinces in Korea and our results were consistent with those of previous studies [6]. Finally, in our study, the PV assay was used to measure the NTAb titers, while PRNT were used in other previous studies [14]. However, a close correlation between the results obtained with the PV assay and the PRNT have been published [15] and validated in our study. Therefore, it is unlikely that the PV assay used in this study would generate different results from those used in previous studies.

## 5. Conclusions

In conclusion, NTAbs to JEV has been maintained at appropriate levels among the general population in Korea. As a result of the study, we propose that adult immunization and a third booster vaccination against JEV are not strongly recommended. Periodic nationwide surveillance on the prevalence of JE NTAb and continuous monitoring for JE cases is required to understand the population-level immunity to JE and determine a proper immunization schedule in Korea.

## Figures and Tables

**Table 1:** Age and geographical distribution of the study population.

C	Provinces					
	Seoul	Wonju	Incheon	Jeonju	Daejeon	Total
	N = 384	N = 280	N = 322	N = 302	N = 315	N = 1603
15 years	24	20	20	21	18	103
F	12	10	4	11	9	46
M	12	10	16	10	9	57
16 years	24	20	20	22	17	103
F	12	10	10	10	8	50
M	12	10	10	12	9	53
17 years	24	20	20	23	19	106
F	12	10	9	10	9	50
M	12	10	11	13	10	56
18 years	24	20	20	23	19	106
F	12	10	5	11	10	48
M	12	10	15	12	9	58
19 years	24	20	20	22	23	109
F	12	10	9	12	11	54
M	12	10	11	10	12	55
20–24 years	24	20	20	25	20	109
F	12	10	4	10	10	46
M	12	10	16	15	10	63
25–29 years	24	20	20	20	20	104
F	12	10	5	10	10	47
M	12	10	15	10	10	57
30–34 years	24	20	20	20	20	104
F	12	10	11	10	10	53
M	12	10	9	10	10	51
35–39 years	24	20	20	20	20	104
F	12	10	10	10	10	52
M	12	10	10	10	10	52
40–44 years	24	20	20	20	20	104
F	12	10	9	10	10	51
M	12	10	11	10	10	53
45–49 years	24	20	20	20	19	103
F	12	10	10	10	9	51
M	12	10	10	10	10	52
50–54 years	24	20	20	20	20	104
F	12	10	10	10	10	52
M	12	10	10	10	10	52
55–59 years	25	20	20	20	20	105
F	12	10	13	10	10	55
M	13	10	7	10	10	50
60–64 years	24	8	20	6	21	79
F	12	3	8	3	10	36
M	12	5	12	3	11	43
65–69 years	23	2	20	9	19	73
F	12	2	9	4	10	37
M	11	0	11	5	9	36
≥70 years	24	10	22	11	20	87
F	12	5	10	5	10	42
M	12	5	12	6	10	45
Total	384	280	322	302	315	1603
F	192	140	136	146	156	770
M	192	140	186	156	159	833

N, number of participants; F, female; M, male.

**Table 2:** Geographical differences of neutralizing antibody titers against the Japanese encephalitis virus.

Age (Years)	Seoul	Wonju	Incheon	Jeonju	Daejeon
15–19					
Number of participants	120	100	100	111	96
Mean NTAb titer	1424.51	923.92	822.68	820.33	947.22
(95% CI)	(1250.43, 1622.82)	(758.26, 1125.77)	(690.26, 980.52)	(664.26, 1013.07)	(756.54, 1185.95)
20–44					
Number of participants	120	100	100	105	100
Mean NTAb titer	871.94	1009.5	229.8	537.13	465.45
(95% CI)	(711.68, 1068.29)	(885.24, 1151.77)	(173.53, 304.30)	(413.71, 697.37)	(365.29, 592.45)
≥45					
Number of participants	144	80	122	86	119
Mean NTAb titer	429.26	402.81	193.12	111.99	130.24
(95% CI)	(340.38, 541.36)	(318.22, 509.89)	(151.30, 246.49)	(86.64, 144.77)	(105.43, 160.89)

NTAb, neutralizing antibody; CI, confidence interval.

## Supplementary Materials

The following are available online at https://www.mdpi.com/2076-393X/8/2/328/s1, Figure S1: Seropositive rates of neutralizing antibody to Japanese encephalitis using plaque reduction neutralization test.
