# Supplementary figures and images for: Seroprevalence of Neutralizing Antibodies against Japanese Encephalitis Virus among Adolescents and Adults in Korea: A Prospective Multicenter Study
Source: Vaccines (Basel). 2020 Jun 19;8(2):328. doi: 10.3390/vaccines8020328 (PMC7350244; doi:10.3390/vaccines8020328)

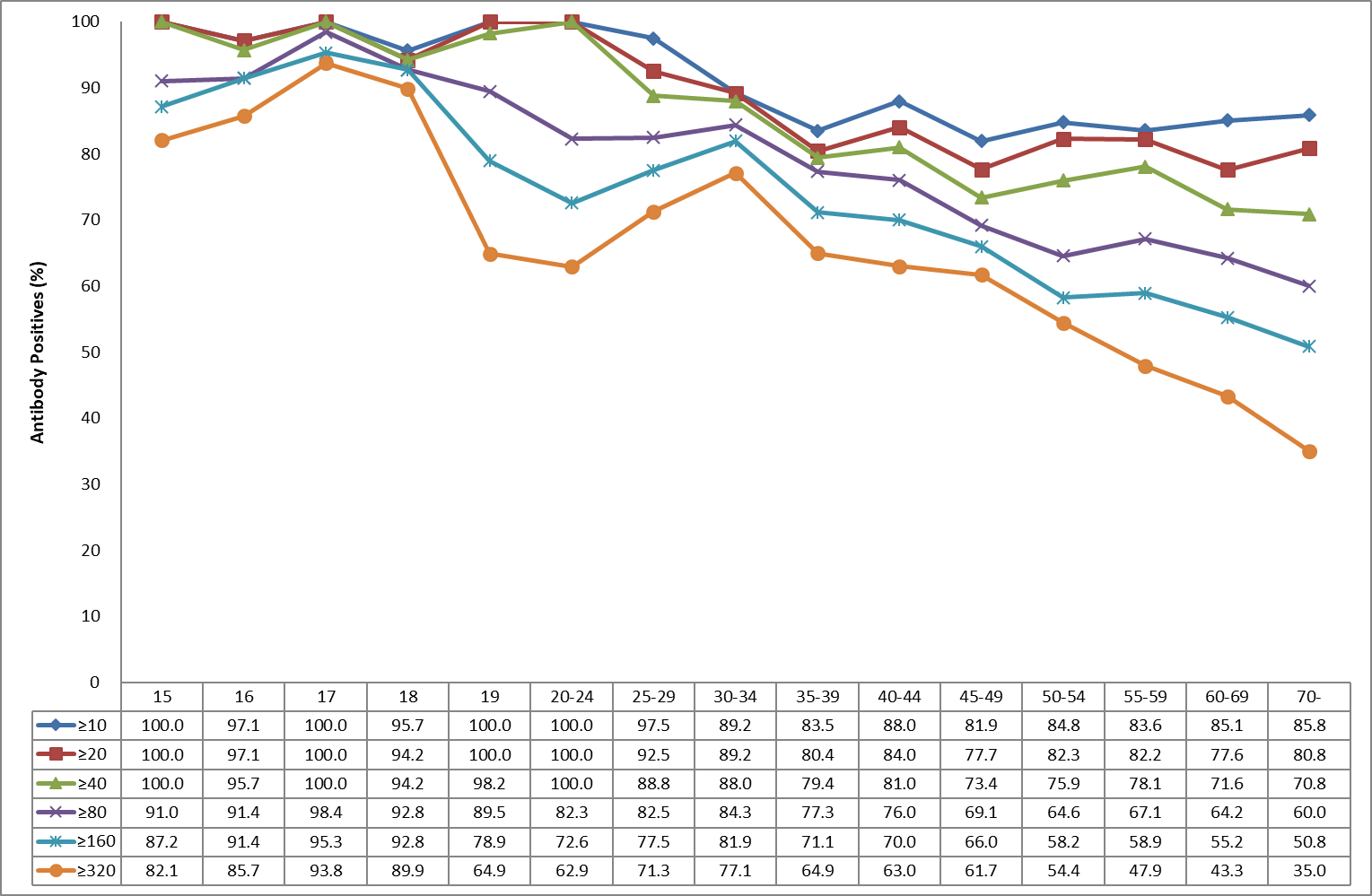

Supplement: Supplementary file 1 [file vaccines-08-00328-s001.zip › vaccines-826041-supplementary.tif]
